# Light-evoked hyperpolarization and silencing of neurons by conjugated polymers

**DOI:** 10.1038/srep22718

**Published:** 2016-03-04

**Authors:** Paul Feyen, Elisabetta Colombo, Duco Endeman, Mattia Nova, Lucia Laudato, Nicola Martino, Maria Rosa Antognazza, Guglielmo Lanzani, Fabio Benfenati, Diego Ghezzi

**Affiliations:** 1 Center for Synaptic Neuroscience and Technology, Istituto Italiano di Tecnologia, Largo Rosanna Benzi 10, 16132 Genova, Italy; 2 Center for Nano Science and Technology, Istituto Italiano di Tecnologia, Via Pascoli 70/3, 20133 Milano, Italy; 3 Dipartimento di Fisica, Politecnico di Milano, Piazza Leonardo Da Vinci 32, 20133 Milano, Italy; 4 Department of Experimental Medicine, University of Genova, Viale Benedetto XV 3, 16132 Genova, Italy

## Abstract

The ability to control and modulate the action potential firing in neurons represents a powerful tool for neuroscience research and clinical applications. While neuronal excitation has been achieved with many tools, including electrical and optical stimulation, hyperpolarization and neuronal inhibition are typically obtained through patch-clamp or optogenetic manipulations. Here we report the use of conjugated polymer films interfaced with neurons for inducing a light-mediated inhibition of their electrical activity. We show that prolonged illumination of the interface triggers a sustained hyperpolarization of the neuronal membrane that significantly reduces both spontaneous and evoked action potential firing. We demonstrate that the polymeric interface can be activated by either visible or infrared light and is capable of modulating neuronal activity in brain slices and explanted retinas. These findings prove the ability of conjugated polymers to tune neuronal firing and suggest their potential application for the *in-vivo* modulation of neuronal activity.

In organic bionics^1^ and bioelectronics^2^ organic conductors or semiconductors are intimately integrated with biological tissue to obtain a targeted functional outcome. In the past ten years, conjugated polymers have been exploited in several applications, such as scaffolds for cell adhesion and growth in regenerative medicine^3^,^4^,^5^, functional layers to improve the cell/electrode interfaces^6^,^7^,^8^ as well as prosthetic devices including implantable electrodes^9^,^10^, cochlear implants^11^ and retinal prostheses^12^,^13^. In these fields, particular emphasis has been devoted to the interface with excitable cells^14^,^15^,^16^,^17^,^18^, such as neurons. Several studies demonstrated the possibility to grow primary neurons^19^, primary astrocytes^20^, stem cells^21^, and different types of cell lines^22^ onto conjugated polymers without altering their viability and/or their functional properties, underscoring the high biocompatibility of these materials.

In recent years, the material sciences have focused a great research effort into engineering materials for the development of new probes and devices for neuronal stimulation^15^,^23^. Neuronal excitation has been extensively proved via planar or protruding electrodes^24^, silicon wafers^25^,^26^, nanostructures^23^, and conjugated polymers. However, the inhibition of neuronal activity has been mostly achieved by using optogenetic methods^27^,^28^, and very few reports exist for planar devices and structures made by functional materials^15^,^29^,^30^. In a recent report, it was shown that the prolonged illumination of conjugated polymers, coated over glass substrates, causes the hyperpolarization of Human Embryonic Kidney (HEK293) cells grown on their surface^31^. Hyperpolarization occurred after a transient depolarization phase attributable to a temperature-induced increase in membrane capacitance. The hyperpolarization itself was associated with an increase in membrane conductance and, at light offset, a rebound decrease in membrane capacitance. These effects were attributable to a localized thermal effect, and could be replicated by non-conducting materials with comparable optical absorption.

Here, we describe the use of organic thin films made of prototypical conjugated polymers, such as regioregular poly(3-hexylthiophene-2,5-diyl) (P3HT) and poly[2,6-(4,4-bis-(2-ethylhexyl)-4H-cyclopenta [2,1-b;3,4-b′]dithiophene)-alt-4,7(2,1,3-benzothiadiazole)] (PCPDTBT) or blends thereof with [6,6]phenyl-C61-butyric-acid-methyl ester (PCBM) for investigating their modulatory effects on the electrical activity of primary hippocampal neurons, explanted retinas, and brain slices. We found that long (500 ms) pulses of light are able to hyperpolarize primary neurons and silence both spontaneous and electrically elicited action potentials. We also found that the same effect occurs in intact nervous tissue, such as explanted retinas and hippocampal slices and that, using appropriate conjugated polymers, firing modulation can be achieved with near-infrared (NIR) illumination. Our results introduce conjugated polymer thin films as a new tool for the photo-inhibition of neuronal activity and propose their potential for *in-vivo* application in pathologies characterized by neural hyperactivity, such as epilepsy, or as retina prosthesis for the rescue of light sensitivity in retina degeneration.

## Results

In this work we utilized the interface between conjugated polymers and live cells or tissues to optically control neural activity. The interface we employed is composed of a pristine polymer layer spin coated on top of a glass substrate. Out of a very large number of conjugated polymers, with distinct spectral properties, we primarily used P3HT. This is a prototypical polymer for optoelectronic applications, that has a large excitation spectrum in the visible range centered in the green region, and has been used in previous studies for cellular excitation^12^,^14^. We also tested PCPDTBT, a low band gap polymer with photosensitivity in the near-infrared (NIR) range, which allows better tissue penetration of the light stimulus^32^.

### Light-induced silencing of evoked neuronal firing in primary hippocampal neurons

We have previously shown^31^ that in HEK293 plated onto P3HT, light stimuli have a biphasic effect on the membrane potential. Indeed, with prolonged light stimuli, an initial depolarization was followed by a long-lasting membrane hyperpolarization (Suppl. Fig. 1a,b). Under these conditions, the depolarization peak was on average at 40.22 ± 4.51 ms after the onset of the light stimulus and the membrane potential returned to the resting value in 123.7 ± 20.7 ms (*n* = 17). This indicates that shorter pulses induce a pure depolarization, while longer pulses induce hyperpolarization. We then sought to investigate if, and to which extent, the hyperpolarizing response to prolonged illumination can be obtained in primary hippocampal neurons plated onto P3HT-coated glass, and whether such effects are sufficient to modulate the firing activity of neurons.

**Figure 1:**
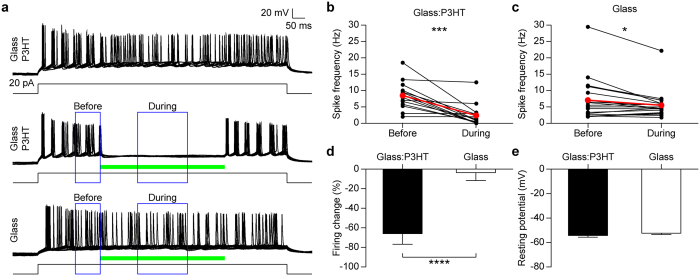
Sustained illumination of primary hippocampal neurons grown on P3HT silences evoked firing. (**a**) A train of action potentials is induced by current injection (20 pA for 1 s; black line) in a representative neuron cultured over glass:P3HT (upper panel). In the same neuron (middle panel), the evoked activity was fully silenced by illumination of the conjugated polymer (500 ms, 16 mW/mm^2^; green bar). Light did not affect the firing activity evoked by current injection in neurons cultured over bare glass (lower panel). Traces are overlays of 10 consecutive sweeps. Blue boxes indicate the time windows used for quantification of the activity before and during the light stimulus. (**b**,**c**) Individual (black symbols) and mean (red symbols) firing rates measured in neurons grown onto glass:P3HT ((**b**); ***p < 0.001, paired *t*-test) or bare glass ((**c**); p = 0.0531, paired *t*-test) before and during the light pulse. (**d**) Mean (±sem) percent firing changes during illumination (negative values for reduction) with respect to the basal firing obtained in the two experimental groups (glass:P3HT, −65.79 ± 10.89%, *n* = 14; glass, −2.10 ± 8.38%, *n* = 19; ****p < 0.0001, *t*-test). (**e**) Mean values (±sem) of the resting membrane potential in neurons grown under the two experimental conditions (glass:P3HT, −54.39 ± 1.35 mV; glass, −52.12 ± 1.02 mV; p = 0.1745, Kolmogorov-Smirnov test).

After formation of a mature network (14–22 days after plating), neurons were recorded in whole-cell current clamp mode, while delivering repeated set-steps of current injection (1 s) sufficient to trigger reliable firing activity (Fig. 1a, top). When a light pulse (500 ms, 16 mW/mm^2^) was administered concomitantly with the current injection used to elicit action potential firing, the spiking activity was silenced during the whole illumination period (Fig. 1a middle and Fig. 1b; p < 0.001, paired *t*-test). The observed reduction (−65.8 ± 10.9%, *n* = 14) was statistically significant with respect to neurons grown onto glass substrates (Fig. 1d; p < 0.0001, *t*-test), whose evoked firing frequency was not significantly affected by the light stimulus (Fig. 1a bottom and Fig. 1c). The individual values of current used to trigger neuronal firing, reported in Suppl. Table 1, show that effective neuronal silencing was present in a wide range of injected current values. Resting membrane potential values were not statistically different across these groups (Fig. 1e; p = 0.17, Kolmogorov-Smirnov test).

### Light-induced hyperpolarization inhibits spontaneous firing activity in primary hippocampal neurons

**Figure 2:**
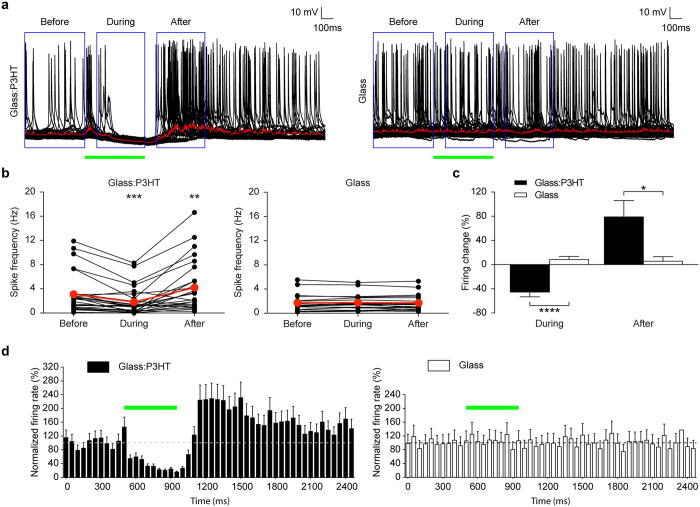
Illumination of primary hippocampal neurons grown on P3HT silences their spontaneous firing activity. (**a**) Spike modulation induced by light stimulation (green horizontal bar) in representative neurons cultured on glass:P3HT (*left*) or bare glass (*right*). Traces are overlays of 63 (left) and 35 (right) consecutive sweeps. The red traces represent the average; the blue boxes indicate the time windows used for quantification of the activity before, during and after the light stimulus. (**b**) Individual (black symbols) and mean (red symbols) firing rates measured before, during and after the light pulse in neurons grown onto glass:P3HT (*left panel;* **p < 0.01; ***p < 0.001, paired *t*-test *vs* before) or glass (*right panel;* during p = 0.5937, after p = 0.7689, paired *t*-test *vs* before). (**c**) Mean (±sem) percent firing changes obtained during (glass:P3HT, −45.34 ± 7.91%, *n* = 27; glass, 8.30 ± 5.03%, *n* = 20; ****p < 0.0001, Kolmogorov-Smirnov test) and after (glass:P3HT, 78.82 ± 27.22%, *n* = 27; glass, 5.57 ± 7.46%, *n* = 20; *p < 0.05, Kolmogorov-Smirnov test) illumination. (**d**) The averaged temporal profiles (mean ± sem) of the responses of the recorded neurons on glass:P3HT or glass are shown as PSTH (bins of 50 ms). Black bars: neurons on Glass:P3HT showing a statistically significant decrease in firing during the light pulse (*n* = 15); white bars: all neurons on bare glass (*n* = 14). The horizontal broken line represents the average baseline value, the horizontal green bar represents the light stimulus.

Given the capability of the polymeric interface to silence the evoked firing activity in response to prolonged illumination, we moved to modulating the spontaneous activity of neurons, thus interfering with their physiological processes. The firing frequency was measured for each neuron, under current clamp conditions, before, during and after illumination (500 ms, 16 mW/mm^2^). The comparison of the average pre-stimulus firing rate to the firing rate during the 100 to 500 ms period of continuous illumination (blue boxes in Fig. 2a) revealed a significant reduction of the spontaneous firing rate on P3HT-coated glass (Fig. 2a,b left panels; p < 0.001, paired *t*-test). The first 100 ms of illumination were excluded from the window since in this time period the optical excitation of the polymer induces depolarization of the membrane (see red traces in Suppl. Fig. 1a,b for HEK293 cells and Fig. 2a for neurons). The light stimulation had no effect on the spontaneous activity of neurons cultured on bare glass (Fig. 2a,b right panels; p = 0.59, paired *t*-test), confirming that the effect is generated by the presence of the polymeric film and that the sustained light stimulation does not induce *per se* any photo-damage. The reduction of spontaneous firing obtained in neurons cultured over P3HT-covered glass substrates during illumination (−45.3 ± 7.9%, *n* = 27) was statistically significant (Fig. 2c; p < 0.0001, Kolmogorov-Smirnov test) with respect to control neurons grown onto glass substrates (8.3 ± 5.0%, *n* = 20). A significant decrease in firing activity was still present at 10.7 mW/mm^2^, but was absent at lower intensities (Suppl. Fig. 2a,b). Under all conditions tested, the resting membrane potential values were not statistically different across the experimental groups (Suppl. Fig. 3; p = 0.13, one-way ANOVA).

To analyze the extent of the light-induced hyperpolarization, the fluctuation in the resting potential was measured after averaging the sweeps recorded for each neuron (red traces in Fig. 2a), effectively filtering out action potentials. This analysis yielded a average amplitude of hyperpolarization of −5.74 ± 0.71 mV (*n* = 27). Consistent with the firing data, the amplitude of neuronal hyperpolarization on P3HT-coated glass was significantly higher than that of control neurons on bare glass exposed to the same light stimulus (−1.08 ± 0.10 mV, *n* = 14; p < 0.0001, Kruskal-Wallis test).

At the light offset, the prolonged hyperpolarization induced by the illumination in neurons grown onto P3HT triggered a rebound increase in the spontaneous firing rate (Fig. 2a left). The spiking frequency after the pulse was evaluated in the 100–500 ms window after the light offset (blue box in Fig. 2a,b), excluding the first 100 ms after the pulse in which a transient rebound hyperpolarization occurs, as visible from the average trace (Fig. 2a left, red trace) and similar to the recordings performed in HEK293 cells (Suppl. Fig. 1b). The comparison of the average firing rate before and after the illumination revealed a significant increase in the firing rate of neurons cultured over P3HT-coated glass substrates (Fig. 2a,b left; p < 0.01, paired *t*-test) and no effect in neurons cultured over bare glass (Fig. 2a,b right; p = 0.76, paired *t*-test). The average firing increase obtained in neurons cultured over P3HT (78.8 ± 27.2%, *n* = 27) was statistically significant (Fig. 2c; p < 0.05, Kolmogorov-Smirnov test) with respect to neurons grown onto glass (5.6 ± 7.5%, *n* = 20). Furthermore, when hyperpolarization was induced by a lower illumination power (10.7 mW/mm^2^), the rebound increase in firing activity was not present (Suppl. Fig. 2a; *n* = 13; p = 0.49, paired *t*-test), indicating the possibility of removing the rebound hyperactivity, while preserving the hyperpolarization response with a proper tuning of the light intensity. The Peri-Stimulus Time Histograms (PSTH), calculated over the entire recording period, clearly show the temporal profile of the firing reduction during the stimulus and the firing increase at the light offset for neurons grown over P3HT (Fig. 2d left), as compared with neurons grown on glass (Fig. 2d right), whose activity was unaffected during and after the light stimulus.

**Figure 3:**
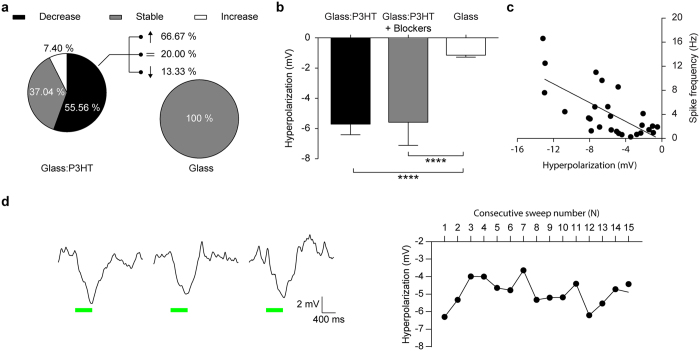
Light stimulation of primary hippocampal neurons grown on P3HT induces membrane hyperpolarization. (**a**) Analysis of the distribution of neurons grown on either glass:P3HT or glass and responding with a decreased, increased or stable firing rate during the light pulse (Glass:P3HT *vs* Glass: decreasing 55.56% *vs* 0.00%; stable 37.04% *vs* 100.00%, increasing 7.40% *vs* 0.00%). The percentage of cell showing a decrease during the pulse on glass:P3HT is statistically significant with respect to those on glass (p < 0.0001, Fisher’s test). For neurons on glass:P3HT showing a decrease in the firing rate during the light pulse, the distribution of the response after the pulse is also described as part of the whole (decreasing 13.33%; stable 20.00%, increasing 66.67%). (**b**) Mean (±sem) hyperpolarization amplitude recorded in neurons grown on glass:P3HT (grey and black bars) or glass (white bar) in the presence of the synaptic blockers AP5/CNQX/PTX (glass:P3HT, −5.74 ± 0.71 mV, *n* = 27; glass:P3HT with blockers, −5.62 ± 1.55 mV, *n* = 14; Glass, −1.08 ± 0.10 mV, *n* = 14; Glass:P3HT *vs* Glass ****p < 0.0001, Glass:P3HT Blockers *vs* Glass ****p < 0.0001, Glass:P3HT *vs* Glass:P3HT Blockers p > 0.9999; Kruskal-Wallis test with Dunn’s multiple comparison test). (**c**) Linear correlation between the hyperpolarization amplitude during illumination and the firing rate after illumination in individual neurons grown on glass:P3HT (Pearson’s correlation coefficient r = 0.69, *n* = 27; p < 0.001). (**d**) Amplitude of the hyperpolarization response to 15 consecutive stimulations (green bars) performed at 0.1 Hz. Potential traces were averaged over the recorded cells on glass:P3HT (*n* = 27). *Left panel:* full traces for sweeps 1, 2 and 15. *Right panel:* average hyperpolarization amplitude of cells grown onto glass:P3HT over the repeated stimulations (sweeps 1–15).

We next analyzed the percentage of neurons grown onto the two substrates that displayed statistically significant changes in firing rates during the light pulse with respect to the baseline across all the sweeps recorded for each neuron (Fig. 3a). While virtually all neurons plated on glass were not affected by illumination, over half of the neurons grown on P3HT showed a significant reduction during the light phase (55.56%). Out of the latter group, over two thirds (66.7%) of the neurons showed a significant rebound increase of activity after the pulse.

To establish whether the light-induced hyperpolarization is due to an effect of the polymer on the intrinsic excitability of neurons or is rather the result of the activity of inhibitory synapses, a separate set of experiments was carried out in which the specific blockers of NMDA, AMPA/kainate glutamate and GABA_A_ receptors D (−)-2-Amino-5-phosphonopentanoic acid (D-AP5), 6-Cyano-7-nitroquinoxaline-2,3-dione (CNQX) and picrotoxin (PTX), respectively, were applied to the recording medium to silence synaptic transmission and the basal spontaneous activity of the network. The hyperpolarization amplitude (Fig. 3b) in the presence of synaptic blockers (gray bar; −5.62 ± 1.55 mV, *n* = 14) closely resembled that measured in the presence of normal synaptic activity (black bar, −5.74 ± 0.71 mV, *n* = 27), indicating that neuronal hyperpolarization does not result from synaptic effects.

To have more insights into the mechanism of the light-induced hyperpolarization, we evaluated whether the rebound increase in firing observed after the light pulse is correlated with the extent of the hyperpolarization obtained during the pulse and whether the neuronal response was attenuating over repeated light stimuli. A significant correlation was found, at the level of single neurons grown on P3HT, between the hyperpolarization amplitude during illumination (obtained by averaging the multiple sweeps recorded in single neurons) and the spiking frequency after the pulse on neurons cultured on P3HT (Fig. 3c; Pearson’s correlation coefficient = 0.69), suggesting that the rebound firing increase is mediated by hyperpolarization-activated inward currents. Finally, when neurons grown on P3HT were subjected to repeated light pulses, their response was not subjected to adaptation and the amplitude of light-induced hyperpolarization remained substantially constant over up to 15 consecutive light stimuli administered at 0.1 Hz (Fig. 3d).

To investigate the ionic mechanism of the light-induced hyperpolarization, we used HEK293 cells that express passive and voltage-dependent K^+^ channels^33^,^34^. HEK293 cells were stimulated for 1500 ms at 16 mW/mm^2^, and the possible contribution of a K^+^ outward current by ionic substitution experiments was examined. The replacement of K^+^-gluconate (*n* = 17) with CsCl (*n* = 13) in the intracellular solution led to a marked reduction (52.6%) of the hyperpolarization amplitude (Suppl. Fig. 1c; p < 0.05, Kruskal-Wallis test with Dunn’s multiple comparison test). Importantly, this reduction was virtually absent when the intracellular solution contained KCl (*n* = 10) or a K^ + ^-gluconate/KCl mixture (*n* = 12) (p > 0.99, Kruskal-Wallis test with Dunn’s multiple comparison test). Resting membrane potential values of HEK293 cells were not statistically different across these groups (Suppl. Fig. 1d; p = 0.35, one-way ANOVA). These data indicate that the hyperpolarization elicited by polymer illumination, at least in HEK293, is largely mediated by endogenous K^+^ conductances.

### Light-induced inhibition of neural activity in acute mouse hippocampal slices

**Figure 4:**
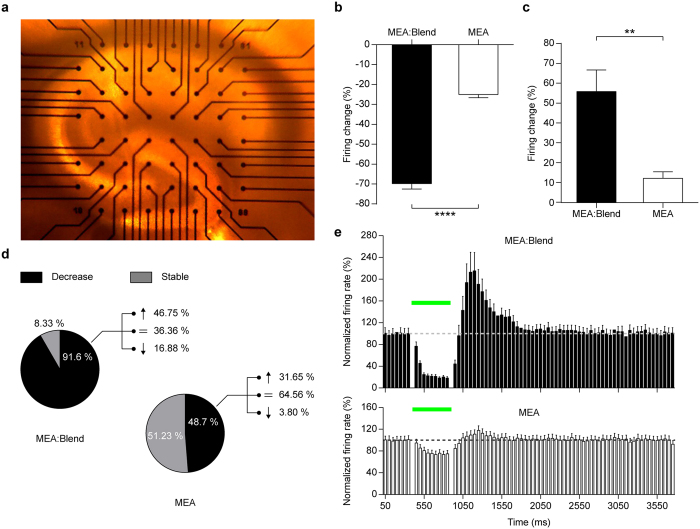
Green light stimulation of acute hippocampal slices on MEAs inhibits the high K^ + ^-evoked firing activity. (**a**) Representative image of an acute cortico-hippocampal slice on MEA. (**b**,**c**) Mean (±sem) percent firing changes during (**b**) and after (**c**) the light pulse (500 ms, 30 mW/mm^2^) from all cells recorded in slices placed on MEA:blend or bare MEA (MEA:blend, during −69.77 ± 2.75%, after 56.03 ± 10.87%, *n* = 84; bare MEA, during −25.06 ± 1.62%, after 12.35 ± 3.22%, *n* = 162; **p < 0.01, ****p < 0.0001, Mann-Whitney U test). (**d**) Pie charts representing the activity distribution of neurons in slices layered onto MEA:blend or bare MEA whose firing displayed a statistically significant decrease (black), increase (white) or remained stable (grey) during illumination (MEA:blend *vs* MEA: decreasing 91.67% *vs* 48.77%; stable 8.33% *vs* 51.23%, increasing 0.00% *vs* 0.00%). The percentage of cell showing a decrease during the pulse on MEA:Blend is statistically significant with respect of those on MEA (p < 0.001, Fisher’s test). For the cell showing a decrease in the firing rate during the light pulse, the distribution after the pulse is also described as part of the whole (MEA:blend *vs* MEA: decreasing 16.88% *vs* 3.8%; stable 36.36% *vs* 64.56%, increasing 46.58% *vs* 31.64%). (**e**) The averaged temporal profiles (mean ± sem) of the responses of the neurons on glass:P3HT or glass are shown as PSTH (bins of 50 ms). Black bars: neurons recorded on MEA:blend showing a statistical decrease of the firing activity during the pulse (*n* = 77); white bars: neurons recorded on bare MEAs (*n* = 162). The horizontal broken line represents the average baseline value, the horizontal green bar represents the light stimulus.

Having established a functional role of our polymeric interface in reducing the firing activity in primary neurons, we sought to translate our results on mouse hippocampal slices under epileptic activity induced by high K^+^ extracellular concentration. First, we layered hippocampal slices on MEAs coated with a blend of P3HT and PCBM (MEA:Blend), to endow the polymeric layer with a proper conductivity for signal acquisition (Fig. 4a).

Similar to cultured primary neurons, the firing activity was significantly reduced during illumination (500 ms, 30 mW/mm^2^) on MEA:Blend (Fig. 4b;−69.8 ± 2.7%, *n* = 84) with respect to bare MEA (p < 0.0001, Mann-Whitney U test). Also the illumination of the polymeric interface triggered a significant rebound increase (Fig. 4c;56.0 ± 10.9%, *n* = 84) of the spiking activity after the stimulus in brain slices on MEA:Blend compared to slices on bare MEA (p < 0.01, Mann-Whitney U test). Next, we classified sorted neurons according to their behavior during illumination with respect to the pre-pulse firing rate, as decreasing, increasing or stable, (Fig. 4d). The population of neurons with decreased activity during illumination was significantly larger in slices on MEA:Blend than in parallel slices on bare MEA (MEA:Blend *vs* MEA; 91.6% *vs* 48.7%, p < 0.001, Fisher’s test). Moreover, while the relative majority of neurons on MEA:Blend that were silenced during the light stimulus displayed an increased firing rate (46.7%) after the light stimulus, the majority of neurons on bare MEA maintained a stable spiking activity (64.5%).

Finally, we compared the PSTHs of the neurons sorted from slices on MEA:Blend that exhibited a significant decrease during the light pulse (*n* = 77) with the PSTHs of the overall recorded cells on bare MEAs (*n* = 162) (Fig. 4e). This analysis revealed that the presence of P3HT:PCBM induced a strong and significant modulation of the firing activity with a marked inhibition during illumination and a rebound firing increase after illumination lasting approximately 700 ms before recovery to baseline, while the corresponding fluctuations in firing activity of slices on bare MEA were slight and not significant. All analyses were carried out on the same time-windows described in the previous section, thereby excluding the light-generated artifacts of the electrodes (Suppl. Fig. 4).

### P3HT mediated inhibition in hippocampal neurons is not dependent upon TRPV activation

**Figure 5:**
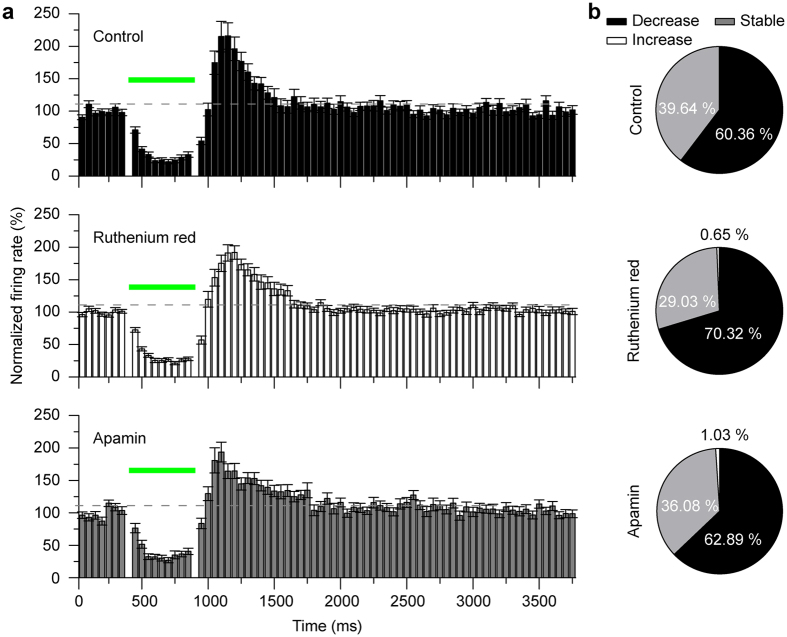
Light-evoked firing activity of mouse hippocampal slices on MEA:blend upon pharmacological blockage of TRPV or SK ion channels. (**a**) The averaged temporal profiles (mean±sem) of the responses, upon green light pulses (50 ms, 28 mW/mm^2^) in different pharmacological conditions, are shown as PSTH (bins of 50 ms). Black bars: neurons recorded in high K^+^ solution showing a decrease of the firing activity during the pulse (*n* = 76); white bars: neurons recorded in high K^+^ solution with the addition of 10 μM Ruthenium Red, a non-specific blocker of the TRPV family temperature-gated ions channels (*n* = 112); gray bars: neurons’ activity in high K^+^ solution in presence of 100 nM Apamin, that blocks SK channels, a type of Ca^2+^-activated K^+^ channel channels (*n* = 61). Both blockades show no significant effect on the percentage firing change during and after the light stimulation. (**b**) The cell population having a significant decrease in activity during the pulse revealed comparable between the three groups (control: 60.36%, Ruthenium red: 70.32%, apamin: 62.89%), thus excluding the hypothesis of a potential TRPV or SK channels’ influence on the light-evoked behavior.

Given that our device generates local and transient increases of temperature, we investigated whether and to what extent the temperature-gated channels of the Transient Receptor Potential Vanilloid (TRPV) channel family play a role in the observed effects^35^. Previous studies utilizing short IR light pulses described laser-evoked hyperpolarization, attributed to Ca^2+^ influx via TRPV channels, and subsequent Ca^2+^-dependent potassium channel activation in sensory neurons^36^. Similar IR-mediated photo-thermal activations of TRPV have also been reported in the vagus nerve^37^, as well as in Drosophila photoreceptors^38^. Given such reports, we carried out a set of experiments to determine the potential contribution of TRPV channels in the P3HT-driven inhibitions described above. Primary hippocampal neurons were exposed to the light stimulation protocols as before in the presence or absence of 10 μM Ruthenium Red (a TRPV non-specific blocker). The inhibition of the spiking activity in response to the light stimulus was not affected by the TRPV channel blocker (Suppl. Fig. 5). Next, we analyzed the effects of Ruthenium Red and Apamin (an SK-channel blocker) on the spike silencing in acute hippocampal slices. Neither the addition of neither 10 μM Ruthenium Red or 100 nM Apamin to the high K^+^ solutions blocked the light activated inhibition both in terms of percentage of neurons displaying reduction and in the extent of firing inhibition (Fig. 5).

### Modulation of firing activity of mouse hippocampal slices can be obtained with near-infrared illumination

**Figure 6:**
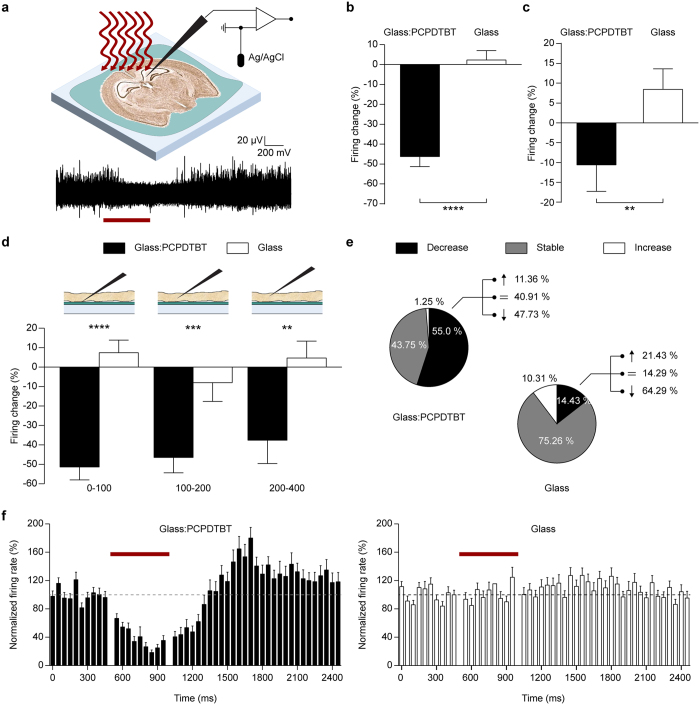
K^+^-evoked firing activity of hippocampal slices can be inhibited by NIR stimulation. (**a**) Experimental set-up and representative trace (overlay of 30 sweeps) under illumination (red bar; 780 nm, 500 ms, 24 mW/mm^2^). (**b**,**c**) Mean (±sem) percent firing changes during (**b**) and after (**c**) illumination. During: Glass:PCPDTBT, −46.44 ± 5.11%, *n* = 80; Glass, 2.27 ± 4.73%, *n* = 97; ****p < 0.0001. After: Glass:PCPDTBT, −10.60 ± 6.67%, *n* = 80; Glass, 8.44 ± 5.18%, *n* = 97; **p < 0.01, Mann-Whitney U test. (**d**) Mean (±sem) percent firing changes during illumination for three distance ranges of the electrode from the PCPDTBT or glass surface (0–100 μm: Glass:PCPDTBT, −51.82 ± 6.74%, *n* = 36; Glass, 5.24 ± 6.57%, *n* = 38; ****p < 0.0001. 100–200 μm: Glass:PCPDTBT, −46.92 ± 7.97%, *n* = 20 Glass, −7.07 ± 9.84%, *n* = 27; ***p < 0.001. 200–400 μm; Glass:PCPDTBT, −37.96 ± 12.08%, *n* = 24 Glass, 4.76 ± 8.73%, *n* = 32, **p < 0.01. Kolmogorov-Smirnov test). (**e**) Distribution of neurons, layered onto Glass:PCPDTBT or glass, showing a statistically significant decrease (black), increase (white) or remained stable (grey) during illumination (Glass:PCPDTBT *vs* Glass: decreasing 55.00% *vs* 14.43%; stable 43.75% *vs* 75.26%; increasing 1.25% *vs* 10.31%). The percentage of cell showing a decrease on Glass: PCPDTBT is statistically significant with respect of those on Glass (p < 0.001, Fisher’s test). For cells showing a decrease during illumination, the distribution after the pulse is reported as part of the whole (Glass: PCPDTBT *vs* Glass: decreasing 47.73% *vs* 64.29%; stable 40.91% *vs* 14.29%; increasing 11.36% *vs* 21.43%). (**f**) PSTH (bins 50 ms, mean ± sem) of neurons on glass:PCPDTBT or glass. Neurons on Glass:PCPDTBT showed a significant decrease of the firing activity during the pulse (*n* = 44, black bars) with respect to those on Glass (*n* = 97, white bars). The horizontal broken line represents the average baseline value, the horizontal red bar represents the NIR stimulation.

To demonstrate the potentiality of photo-inhibition by the polymeric interface at various wavelength of incident light, we performed a second series of experiments by using a polymer with photosensitivity in the NIR range, namely PCPDTBT, which could in principle extend the present technique to applications requiring depth penetration of light, such as *in-vivo* optical stimulation. Experiments with sharp metal electrodes were therefore performed under 780 nm illumination (500 ms, 24 mW/mm^2^) on acute hippocampal slices placed over PCPDTBT-coated glass substrates (Fig. 6a), with induced epileptic activity brought on by high extracellular K^+^. The spiking activity was recorded from the slices before during and after the illumination as described above. Interestingly, the mean firing change of all the neurons recorded over PCPDTBT showed a sustained and highly significant reduction of activity during the light pulse (Fig. 6b;−46.4 ± 5.1%, *n* = 80) as compared with parallel recordings from slices on bare glass (2.2 ± 4.7%, *n* = 97; p < 0.0001, Mann-Whitney U test). However, in contrast with previous results, the silencing lasted also after the light stimulus was switched off (Fig. 6c;−10.6 ± 6.6%, *n* = 80) and the rebound hyperactivity was delayed and modest (500 to 900 ms after light offset: 41.8 ± 7.6%). As expected, the firing changes in neurons on glass during and after illumination were negligible and not significantly different from zero (Fig. 6b,c).

Once the reproducibility of the technique regardless of the change of material and illumination wavelength was proven, we quantitatively analyzed the PCPDTBT effect by placing the metal electrode at increasing distances from the polymer surface (Fig. 6d). While a progressive decrease in the inhibitory effect was found with increasing distances from the polymer surface, the attenuation of the effect was small and a distance of approximately 400 μm reduced the silencing effect only by 10%, suggesting the possibility to use this interface for *in-vivo* inhibition.

Next, we evaluated the distribution of neurons displaying a significant change in firing rate during illumination (Fig. 6e). More than half the recorded neurons on PCPDTBT showed a decrease in activity (55.0%), with very few neurons revealing the same behavior on glass (14.4%; p < 0.001, Fisher’s test). Finally, we compared the temporal profiles of the response of neurons on PCPDTBT that displayed a significant decrease in firing with those of all the recorded neurons on glass (Fig. 6f). The PSTHs confirmed that the reduction in spiking activity during illumination extended to the post-stimulus phase and the post-illumination rebound hyperactivity was delayed, short-lived and reduced in amplitude.

### Light-induced inhibition of spiking activity of retinal ganglion cell firing in blind retina explants

**Figure 7:**
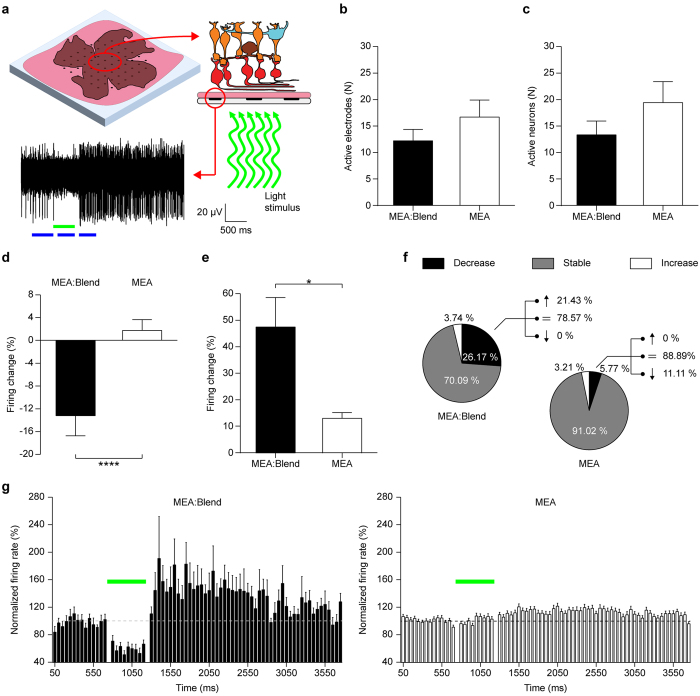
Green illumination of degenerate retinas suppresses the spontaneous activity of ganglion cells. (**a**) Experimental set-up and representative trace (overlay of 20 sweeps) recorded from one electrode upon illumination (green bar; 500 ms, 28 mW/mm^2^). Blue bars represent the analysis windows corresponding to “before”, “during” and “after” illumination. (**b**,**c**) Number of active electrodes (**b**) and neurons (**c**) per experiment. Active electrodes: MEA:blend, 12.25 ± 2.14, *N* = 8; MEA, 16.75 ± 3.20, *N* = 8; p = 0.2617. Active neurons: MEA:blend, 13.38 ± 2.62, *N* = 8; MEA, 19.50 ± 3.94, *N* = 8; p = 0.2161; unpaired *t*-test. (**d**,**e**) Mean (±sem) percent firing change during (**d**) and after (**e**) illumination over all the recorded cells. During: MEA:blend, −13.34 ± 3.53%, *n* = 107; MEA, 1.64 ± 1.94%, *n* = 156; ****p < 0.0001. After: MEA:blend, 47.84 ± 11.16%, *n* = 107; MEA, 13.26 ± 2.19%, *n* = 156; *p < 0.05, Mann-Whitney U test. (**f**) Distribution of neurons in retinas layered onto MEA:blend or MEA showing a statistically significant decrease (black), increase (white) or remained stable (grey) during illumination (MEA:blend *vs* MEA: decreasing 26.17% *vs* 5.77%; stable 70.09% *vs* 91.02%, increasing 3.74% *vs* 3.21%). The percentage of neurons showing a decrease during the pulse on MEA:Blend is statistically significant with respect of those on MEA (p < 0.001, Fisher’s test). For the cell showing a decrease in the firing rate during illumination, the distribution after the pulse is described as part of the whole (MEA:blend *vs* MEA: decreasing 0.00% *vs* 11.11%; stable 78.57% *vs* 88.89%, increasing 21.43% *vs* 0.00%). (**g**) PSTH (bins 50 ms, mean ± sem) of neurons recorded on MEA:blend or MEA. The cells recorded on MEA:blend show a significant decrease in firing activity during the light pulse (*n* = 28, black bars) *versus* all the cells recorded on bare MEAs (*n* = 156, white bars). The horizontal broken line represents the average baseline value, the horizontal green bar represents the light stimulus.

The function of the retina is to translate incident light into a modulation of the firing rate of its output neurons, the retinal ganglion cells (RGCs). Interestingly, the firing modulation obtained in the experiments described above is reminiscent of the OFF response of retinal ganglion cells (RGCs) to light, thus suggesting a potential application as epiretinal prosthetic device. To evaluate this potential application, we applied the same illumination protocol described above (500 ms, 28 mW/mm^2^) to acutely dissected retinas layered on either MEA:Blend or control bare MEA in epiretinal configuration, i.e. with RGCs in contact with the MEA (Fig. 7a). To avoid intrinsic photoreceptor responses to light stimuli and demonstrate the recovery of the light mediated modulation of retinal output, we used retinas with advanced photoreceptor degeneration from adult (3–4 months old) Royal College of Surgeons (RCS) rats, a widely accepted model of *Retinitis pigmentosa*^39^. Moreover, recordings were performed in the presence of the synaptic blockers L-(+)-2-Amino-4-phosphonobutyric acid (L-AP4) and CNQX to further isolate RGCs from the remaining retinal circuitry.

We initially evaluated the coupling of the explant to the device by recording spontaneous firing activity in the dark and comparing retinas on either MEA:Blend or bare MEA for the number of active electrodes and active neurons, after sorting of the multi-unit signals into single-unit activities (Fig. 7b,c). The number of active electrodes and active neurons in retinas on P3HT:PCBM were not statistically different from those recorded with retinas on glass, indicating the polymer coating was not markedly affecting the sensitivity and/or the S/N ratio of the MEA device. When the firing change of all recorded RGCs was analyzed in the same windows as before, a significant silencing of the spiking activity during the illumination was observed in retinas layered on MEA:Blend (Fig. 7d;−13.3 ± 3.5%, *n* = 107), whereas no effect was present in retinas on bare MEA (1.6 ± 1.9%, *n* = 156; p < 0.0001, Mann-Whitney U test).

As observed in primary neurons and acute brain slices, the presence of the polymeric blend triggered rebound hyperactivity after the light pulse (Fig. 7e; 47.8 ± 11.2%, *n* = 107) that was virtually absent in retinas on bare MEA (13.3 ± 2.2%, *n* = 156; p < 0.05, Mann-Whitney U test). The distribution of RGCs that displayed a significant modulation of the firing activity during the light pulse showed that, in both groups, the majority of the cells had a stable frequency (Fig. 7f). Nevertheless, 26% of the RGCs on MEA:blend experienced a silencing of the activity during illumination, while only 5% of the RGC population on bare MEA showed such a decrease (p < 0.001, Fisher’s test). More than 20% of RGCs on MEA:Blend that were silenced during illumination showed hyperactivity after the light pulse. The PSTH analysis of the silenced RGCs on MEA:Blend *versus* the overall recorded RGC population on glass showed that silenced RGCs exhibit a prolonged rebound increase in firing lasting 1–2 sec after the light stimulus, while the PSTH of RGCs on glass is flat, confirming that the RCS retina are insensitive to the applied light stimulus (Fig. 7g).

Finally, to confirm that the observed effects are not due to the presence of PCBM in the polymer coating of the MEA chips, we recorded retina explants onto pristine P3HT-coated glass using sharp metal electrodes. We found that pristine P3HT was as effective as the P3HT:PCBM blend used in the MEA experiments in modulating RGC firing activity in response to illumination (Suppl. Fig. 6), indicating that P3HT can entirely account for the effects of the P3HT:PCBM blend used in the MEA experiments.

## Discussion

In the past few years, conjugated polymers have been used in a variety of optoelectronic devices, including biomedical applications. In particular, we recently exploited such materials in neural interfaces for optical stimulation of cells. We found that illumination with brief (<100 ms) light pulses elicited electrical activation of cultured neuronal networks and of blind retinas layered in sub-retinal configuration, suggesting potential applications in retinal prosthetics to cure degenerative blindness^12^. Measurements in astrocytes also displayed a depolarization of the membrane in response to continuous light illumination that was mediated by an increase in Cl^−^-conductance through ClC-2 channels^20^. An open issue was, however, the response of the polymeric interface to prolonged light stimuli, as those impinging on the retina during normal life. In a recent report, we showed that non-excitable HEK293 cells grown on P3HT-coated glass substrates respond to prolonged illumination with a transient depolarization followed by sustained hyperpolarization. This complex behavior was demonstrated to arise from a local temperature increase due to the intense light absorption by the polymer^31^ affecting both the cell’s membrane capacitance and the electrochemical equilibrium potential. Thus, the cell responses to long light stimuli may differ amongst cell types depending on the interplay between generalized membrane effects and responses mediated by the specific complement of a membrane’s ion channels and transporters. Consequently, it was of primary interest to determine whether sustained illumination would lead to hyperpolarization in neurons, a cell type characterized by a complex ion channel composition.

Here we report that prolonged light stimuli do indeed induce an overall inhibitory effect on neuronal networks at various levels of complexity, from low-density primary cultures to acute brain slices and retina explants. Our results demonstrate the ability of conjugated polymers to act as a reliable interface for inducing a cell-autonomous hyperpolarization, capable of achieving marked silencing of spontaneous and evoked firing activity that could be exploited to control pathological conditions of hyperexcitability. We first documented the nature of the membrane potential response of cultured primary hippocampal neurons. Here we found that illumination leads to a hyperpolarizing response that lasted until the end of the light stimulus, and furthermore that this hyperpolarization preserved its shape and amplitude both over repeated stimulations and in the presence of synaptic blockers. The use of blockers of excitatory and inhibitory synaptic transmission revealed that the observed effect is a cell-autonomous phenomenon and does not result from the light-mediated excitation of inhibitory interneurons. Moreover, when the light stimulus was switched off, the vast majority of previously silenced neurons displayed a rebound hyperexcitability with a corresponding increase in firing that was highly correlated with the amplitude of hyperpolarization under illumination and that is likely to reflect activation of depolarizing conductances at the end of the light stimulus.

Two basic mechanisms acting at the polymer-neuron interface can be hypothesized: electrical and/or thermal. In our previous work in HEK293 cells^31^, the surface potential established at the interface between the polymer and the electrolyte, detected with an electrode in the close proximity of the polymer surface (2 μm), was virtually absent in absence of the ITO electrode, both in the case of pristine polymer and polymer blend.

It is generally accepted that the capacitive charging of the interface requires the presence of an ITO electrode or other anode (hole injection layer) to collect electrons generated by illumination and generate the photo-potential and, accordingly, we observed that charge dissociation preferentially occurs at the ITO/polymer interface (Antognazza and Lanzani, unpublished results). Moreover, the short rise time of the photovoltage in the presence of an ITO electrode (<15 ms) rules out that the generation of photovoltage plays a significant role in the 500 ms continuous illumination during which the cell is hyperpolarized. Given that our interface lacks the ITO electrode, electrical effects, which may depend on polymer thickness, are unlikely to lead to sizable effects on cell membrane properties. The possibility that the polymer releases specific products of photo-electrochemical reactions in the extracellular bath is also unlikely. In fact, faradaic currents are negligible in the absence of ITO electrode, electrical connection and external bias and, even by applying an external bias of few hundreds of mV, neither the optical absorption spectrum nor the Raman spectrum of the polymer are affected by substantial changes (Antognazza and Lanzani, unpublished results).

On the other hand, light absorption by the polymer leads to the generation of different photoexcited states that will recombine non-radiatively to the ground state by locally releasing thermal energy. Such thermal effect can be accurately quantified, showing that heat diffusion in the polymer layer, upon 250 msec illumination, is uniform over a polymer thickness of at least 150 nm (Suppl. Fig. 7). As previously shown^31^, the potential mechanism by which a localized increase in temperature at the interface building up over long stimuli induces hyperpolarization and firing inhibition is likely to be mediated in parallel by (i) activation of membrane conductances (ii) a hyperpolarizing shift of the membrane potential based on the GHK equation, and (iii) a rebound decrease in membrane capacitance at the light offset. This likely thermal mechanism is further bolstered here by the identification of a specific K^+^ conductance activation in HEK293 that is responsible for over half of the amplitude of the observed hyperpolarizing response to illumination. The K^+^ conductance could in principle be activated by calcium entry through TRPV channels. However, our data show that these channels are not involved in the hyperpolarization effect.

We also asked whether the light stimulation might affect cell viability by inducing photo-damage. While high intensity and brief light pulses can cause cell damage, it is unlikely that this possibility applies to our experimental approach, as cells grown in the absence of polymer and illuminated with the same protocol did not show any changes in the resting potential or in the spontaneous firing activity. Moreover, the continuous monitoring of neurons plated on either Glass or Glass:P3HT that were repeatedly stimulated with light at 0.1 Hz always reported hyperpolarization without major changes in their main membrane properties.

The polymer mediated hyperpolarization was not only effective at modulating the activity of neuronal cultures, but also elicited similar effects both in acute brain slices and blind retina explants, indicating a strong translational potential of the films for extracellular inhibition of neural activity. The capability of the interface to silence *in-situ* neurons rendered epileptic by high extracellular K^+^ suggests a potential use of this light-powered interface for antiepileptic therapy. Nevertheless, the achieved results have two major limitations that may hamper this exploitation. First, while the activity silencing would be beneficial, the rebound hyperactivity is detrimental and can trigger further paroxysmal activity. Our initial results do however demonstrate that the rebound activity can be avoided by tuning the light intensity to a level in which it is still able to hyperpolarize but does not elicit rebound firing. The second aspect limiting the *in-vivo* applicability of the interface is related to the need of visible light to excite P3HT. Although this does not represent a problem for epi-cortical implants, the effect will be reduced in case of deep brain stimulation by the low penetration of visible light in the nervous tissue. For this reason, we demonstrated here that an alternative low band gap conjugated polymer, sensitive to the NIR is equally effective in inhibiting neuronal firing. In addition, this polymer shows a substantially reduced hyperactivity at the light offset. Although the reason for such attenuation of the rebound increase in firing is at the moment unknown, the finding represents an important advancement for the generation of biocompatible and flexible devices for the non-invasive inhibition of neuronal activity.

A second potential application demonstrated in this work consists in the modulation of RGC activity in degenerate retinas. Thus, we asked whether this interface could be used to drive the activity in photoreceptor degenerate retinas of adult RCS rats, a model of *Retinitis pigmentosa*. The pattern of modulation we observed in response to visible light qualitatively resembled those of healthy OFF RGCs, extending previous reports that suggested the employment of conjugated polymers as prosthesis to rescue light sensitivity in blind retinas^12^,^13^.

Taken together, the results demonstrate the reliability of the polymeric interface to non-invasively obtain membrane hyperpolarization of neurons and silencing of their firing activity upon illumination in three distinct neural preparations of increasing complexity. Three main strategies have been described for the non-invasive light-mediated inhibition of neuronal activity, namely: opto-pharmacology^40^, optogenetics^41^, and thermal modulation^29^. In recent years, photo-thermal stimulation has gained significant traction in the biomedical field, ranging from modulation of neural activity to targeted thermal treatments for cancer therapy^42^. In this emerging field, models have been recently proposed to explain the multi-phasic response of the membrane potential to thermal stimuli^31^,^43^,^44^. The data presented here contribute to this field and investigate how a stimulation strategy might be exploited for biomedical research, taking advantage of the versatile polymeric interface. In conclusion, the presented technique allows a significant extent of inhibition to be rapidly and repeatedly attained over large areas and with flexible substrates optically tunable to a wide spectral range of activation.

## Methods

### Semiconducting polymers

rr-P3HT (Sigma-Aldrich) has a regio-regularity higher than 95%, a molecular weight between 15 and 45 kg/mol, purity higher than 99.995%. PCPDTBT (1-Material Inc) has a molecular weight ca. 30 kg/mol, a polydispersity index 1.9, purity >98%. PCBM (Nano-C) has a purity 99.5%. All materials were used without further purification. Glass coverslips were rinsed and sonicated in deionized water, acetone and isopropyl alcohol. For patch-clamp and metal electrodes experiments, 1,2-dichlorobenzene solutions of P3HT and PCPDTBT were prepared using a magnetic stirrer at 70 °C diluting to concentrations of 30 g l^−1^ and 28 g l^−1^ respectively. For MEA experiments, separate 1,2-dichlorobenzene solutions of P3HT and PCBM were similarly obtained both at a concentration of 7.5 g l^−1^ and then mixed in a 1:1 volume ratio. Solutions were finally heated to 50 °C for 20 min while stirring and spin-coated onto glass or MEA chips (Multichannel Systems, 60MEA200/30-Ti). Spinning parameters were tuned to obtain suitable optical quality and thickness (P3HT, 230 nm, PCPDTBT, 170 nm, P3HT:PCBM, 170 nm) as follows: 1) P3HT, first step 800 rpm for 3 s, second step 1600 rpm for 60 s; 2) PCPDTBT, first step 800 rpm for 30 s, second step 1600 rpm for 30 s; 3) P3HT:PCBM, first step 800 rpm for 30 s, second step 1600 rpm for 30 s. The polymeric films were finally treated by oxygen plasma to improve the hydrophilicity of the surface (30 s, 10 W). Samples for neuronal cultures were further sterilized at 120 °C for 2 h. Optical absorption spectra (Suppl. Fig. 8) were acquired on thin films deposited on glass coverslips, after thermal annealing (CARY 50 UV-VIS spectrophotometer, VARIAN).

### Cell culture procedures

Human Embryo Kidney (HEK293) cells, obtained from ATCC, were cultured in Dulbecco’s Modified Eagles Medium (DMEM) supplemented with 10% fetal calf serum, 2 mM L-glutamine, 100 μg/ml penicillin, and 100 μg/ml streptomycin (Life Technologies). Cultures were maintained at 37 °C in a humidified atmosphere containing 5% CO_2_. Primary cultures of hippocampal neurons were prepared from embryonic 18-day rat embryos (Charles River). Briefly, hippocampi were dissociated by a 15-min incubation with 0.25% trypsin at 37 °C and cells were plated on poly-L-lysine-coated substrates in Neurobasal supplemented with 2 mM L-glutamine, 2% B27, 100 μg/ml penicillin and 100 μg/ml streptomycin, and with 10% horse serum (Life Technologies) in the first 4 h of plating.

### Tissue preparation

All animal procedures were performed in accordance with the guidelines established by the European Community Council (Directive 2012/63/EU of 22 September 2010) and were approved by the Italian Ministry of Health. Retinas were explanted from RCS rats (age 107 ± 5 days, mean ± sem) after a 1 h dark-adaptation and sacrificed with CO_2_ inhalation followed by cervical dislocation. The eye and retina dissection was realized under dim red light. Eyes were enucleated and transferred to carboxygenated Ames’ medium (Sigma Aldrich). The cornea, lens and vitreous were removed and the retina was detached from the sclera. Finally, retinas were transferred to the microscope stage for recordings. Acute hippocampal slices were prepared from 2–4 months old C57BL/6J mice sacrificed with CO_2_ inhalation. Brains were removed and bathed in a carboxygenated ice-cold solution containing in mM: 2.5 KCl, 10 MgSO_4_, 1.2 NaH_2_PO_4_, 25 NaHCO_3_, 25 D-glucose, and 185 sucrose. Coronal hippocampal slices of 400 μm thickness were cut using a vibratome (Leica VT 1200 S) in the aforementioned solution and immediately transferred for 30 min to carboxygenated ACSF recovery solution at 35 °C (in mM: 119 NaCl, 2.5 KCl, 2 MgSO_4_, 2 CaCl_2_ 1.2 NaH_2_PO_4_, 25 NaHCO_3_, 12.5 D-glucose). Slices were then kept at room temperature for 1 h in ACSF prior to recordings.

### Electrophysiology

Whole-cell patch-clamp recordings of HEK293 (between 24 and 48 h after plating) and primary neurons (between 14 and 21 DIV) were performed at room temperature using borosilicate patch pipettes (3.5–5.0 MΩ) and under GΩ patch seal. The extracellular solution contained (in mM): 135 NaCl, 5.4 KCl, 1 MgCl_2_, 1.8 CaCl_2_, 5 HEPES, 10 glucose adjusted to pH 7.4 with NaOH. The standard intracellular solution contained (in mM): 126 K-Gluconate, 4 NaCl, 2 MgSO_4_, 0.2 CaCl_2_, 0.08 Bapta, 9.45 Glucose, 5 Hepes, 3 ATP, and 0.1 GTP. K^+^ substitution was performed by replacing intracellular K-Gluconate with 126 mM CsCl, and a control solution for the increased Cl^−^ was made by substituting K-Gluconate with 126 mM KCl. Recordings were carried out using either a Multiclamp 200B (Axon Instruments) or an EPC10 (HEKA) amplifier. Responses were amplified, digitized at 20 kHz and stored with either pCLAMP 10 (Axon Instruments) or Patchmaster V2.73 (HEKA Elektroniks). To block excitatory and inhibitory synaptic transmission, recordings were performed in the presence of AP5 (50 μM), CNQX (10 μM) and PTX (100 μM) all from Tocris (USA). Retinal explants were superfused with Ames’ medium (Sigma-Aldrich) supplemented with L-AP4 (20 μM; Tocris, USA) to saturate group III metabotropic glutamate receptors and CNQX (20 μM; Tocris, USA) to block AMPA/kainate glutamate receptors, thus inhibiting residual intrinsic light sensitivity related to rods and cones. Additional 100 μg/ml penicillin and 100 μg/ml streptomycin (Life Technologies) were included in the perfused medium. Hippocampal slices were recorded under constant perfusion of a high K^+^ ACSF (in mM: 111.5 NaCl, 10 KCl, 2 MgSO_4_, 2 CaCl_2_ 1.2 NaH_2_PO_4_, 25 NaHCO_3_, 12.5 D-glucose). To investigate a possible involvement of TRPV and SK channels in the mechanism of cell hyperpolarization and firing reduction, Ruthenium Red (10 μM; Sigma-Aldrich) and Apamin (100 nM; Latoxan) were respectively added to the recording media. All media were constantly gassed with 95% O_2_ and 5% CO_2_ and kept at 34–35 °C. Recordings with sharp metal electrode were acquired using a DAM80 differential amplifier (World Precision Instruments), digitized at 20 kHz with Multiclamp 200B, and filtered between 300 Hz and 3 kHz. Spike detection and sorting were performed using the Matlab-based algorithm Wave_clus (https://vis.caltech.edu/~rodri/Wave_clus/Wave_clus_home.htm). MEA recordings were performed using the MEA1060-inv system (Multi Channel Systems). Data were acquired at 25 kHz and filtered between 200 Hz and 3 kHz. Spike detection and sorting were performed using MC Rack software (Multi Channel Systems).

### Photostimulation

Illumination of HEK293 cells and neurons was carried out on a Nikon FN1 upright microscope (Nikon Instruments) by using a combination of 510 nm and 550 nm wavelengths of Spectra X LED system (Lumencor) to match the P3HT absorption spectrum. The light source had a final power of 16 mW/mm^2^. Sharp metal electrode experiments on retinal explants were carried out using the same upright microscope using a 530 nm LED (Optoled, Cairn Research) at 15 mW/mm^2^. Hippocampal slice recordings on the NIR-sensitive polymer utilized a 780 nm LED (Thorlabs) at 24 mW/mm^2^. Light-triggered extracellular activity in MEA experiments was obtained with a mercury lamp (Nikon Instruments) coupled to a dichroic filter peaking at 540 nm (Semrock) through an inverted Nikon Eclipse Ti microscope (Nikon Instruments). The illumination spot covered an area of about 1 mm^2^ with a power density respectively of 28 mW/mm^2^ and 30 mW/mm^2^ for retina explants’ and brain slices’ experiments.

### Statistical analysis

Statistical tests were selected based on the data distribution. Normal distribution of the data was verified using the D’Agostino and Pearson omnibus normality test (p value = 0.05). The sample size was indicated as number of recorded cells/neurons (*n*) or number of recorded retinal explants (*N*).

## Additional Information

**How to cite this article**: Feyen, P. *et al*. Light-evoked hyperpolarization and silencing of neurons by conjugated polymers. *Sci. Rep.* **6**, 22718; doi: 10.1038/srep22718 (2016).

## Supplementary Material

Supplementary Information
