# Supplementary material for: Light-evoked hyperpolarization and silencing of neurons by conjugated polymers
Source: Sci Rep. 2016 Mar 4;6:22718. doi: 10.1038/srep22718 (PMC4778138; doi:10.1038/srep22718)
Supplement: Supplementary Information [file srep22718-s1.pdf]

Light-evoked hyperpolarization and silencing of neurons by conjugated polymers

Paul Feyen[1,‡], Elisabetta Colombo[1,2,‡], Duco Endeman[1], Mattia Nova[1], Lucia Laudato[2], Nicola Martino[2,3], Maria Rosa Antognazza[2], Guglielmo Lanzani[2,3], Fabio Benfenati[1,4]*, Diego Ghezzi[1]*

[1]Department of Neuroscience and Brain Technologies, Istituto Italiano di Tecnologia, Via Morego 30, 16163 Genova, Italy
[2]Center for Nano Science and Technology, Istituto Italiano di Tecnologia, Via Pascoli 70/3, 20133 Milano, Italy
[3]Dipartimento di Fisica, Politecnico di Milano, Piazza Leonardo Da Vinci 32, 20133 Milano, Italy
[4]Department of Experimental Medicine, University of Genova, Viale Benedetto XV 3, 16132 Genova, Italy

[‡,*] Contributed equally to this work.

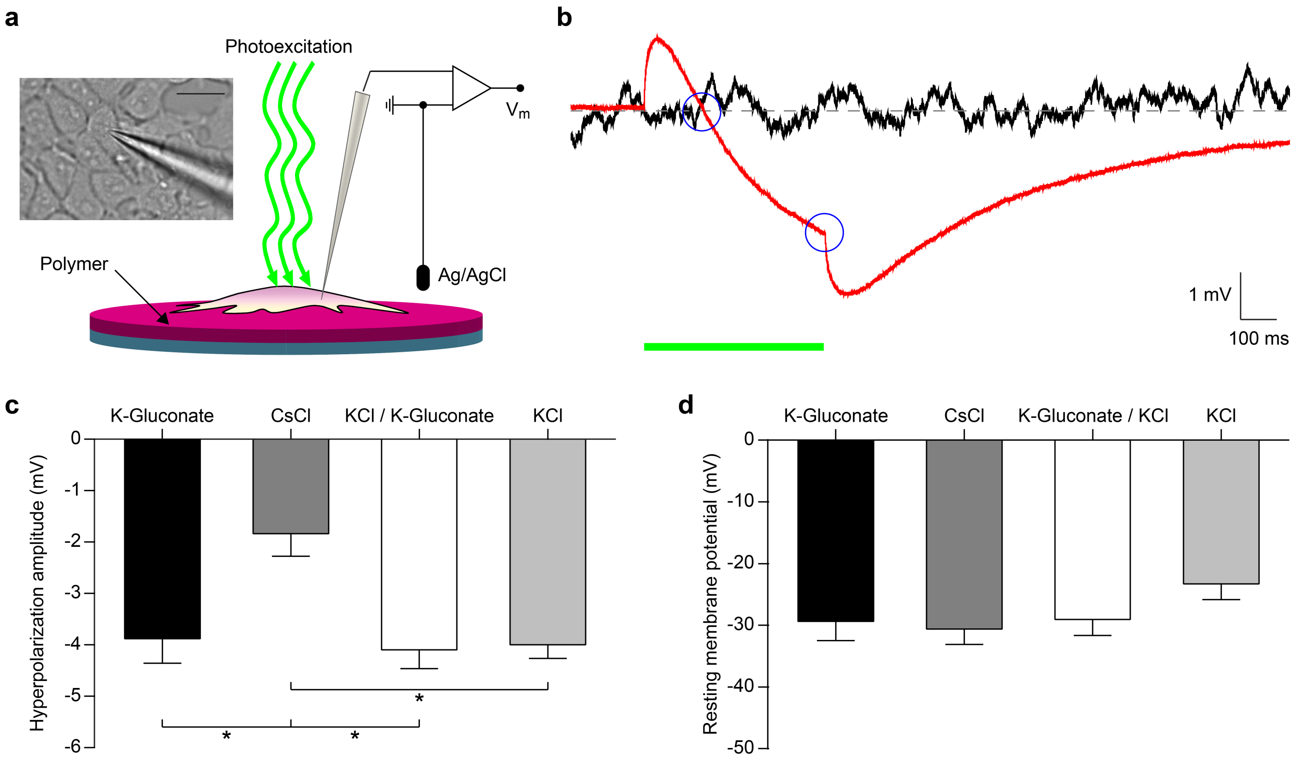

Supplementary Figure 1 | Bimodal modulation of the membrane potential in HEK293 cells.

a. Schematic representation of the experimental set-up showing a HEK293 cell layered over the conjugated polymer P3HT and measured in current-clamp configuration upon illumination. The inset shows an example of patched HEK293 cell. Scale bar, 25 μm.

b. Cell responses to illumination of the polymer with a single pulse of 500 ms (16 mW/mm^2). Representative recordings from a cell over glass:P3HT (red trace) or bare glass (black trace) substrates. The dashed line represents the pre-stimulus resting membrane potential. Blue circles label the depolarization/hyperpolarization transition and the measured hyperpolarization amplitude.

c. Quantification of the maximal hyperpolarization amplitude (means ± sem) in the presence of various intracellular solutions (K-Gluconate, -3.90 ± 0.48 mV, *n* = 17; CsCl, -1.85 ± 0.44 mV, *n* = 13; K-Gluconate/KCl, -4.12 ± 0.37 mV, *n* = 12; KCl, -4.02 ± 0.27 mV, *n* = 10; * $p < 0.05$, Kruskal-Wallis test; Dunn's multiple comparison test).

d. Resting membrane potentials (means ± sem) of HEK293 cells recorded with the following intracellular solutions: K-gluconate (-29.37 ± 3.09 mV, *n* = 17), cesium-chloride (-30.62 ± 2.48 mV, *n* = 13), K-gluconate/KCl (-29.05 ± 2.58 mV, *n* = 12), KCl (-23.28 ± 2.54 mV, *n* = 10). No significant difference among the groups ($p = 0.3556$, one-way ANOVA).

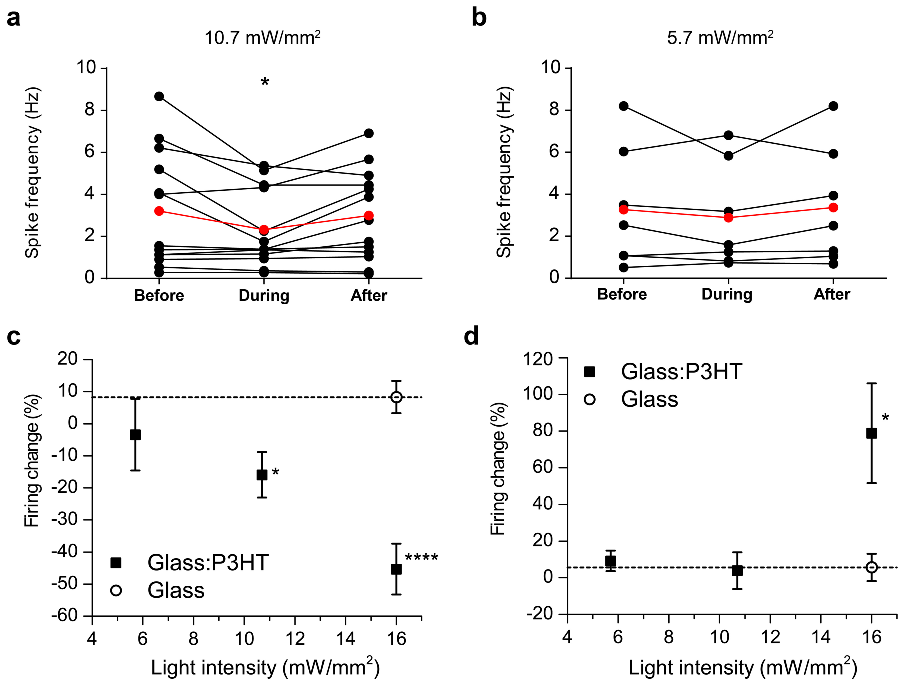

Supplementary Figure 2 | Dose-dependence of neuronal responses to light intensity.

a,**b**. Spike frequencies measured in neurons grown onto glass:P3HT before, during, and after 500ms illumination at 10.7 mW/mm^2 (**a**, n = 13; before p < 0.05, paired *t*-test; after p = 0.4943, paired *t*-test) and 5.7 mW/mm^2 (**b**, n = 7; before p = 0.3759, Wilcoxon matched-pairs signed rank test; after p = 0.4375, Wilcoxon matched-pairs signed rank test).

c,d. Firing changes recorded in neurons during (**c**) and after (**d**) illumination as a function of the stimulation intensity. At the intermediate light power a significant hyperpolarizing response is still present, whereas post-pulse hyperactivity is abolished (* p < 0.05, **** p < 0.0001; unpaired *t*-test).

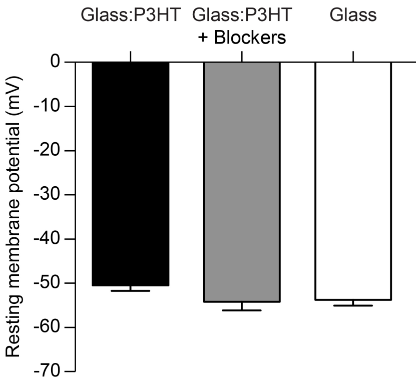

Supplementary Figure 3 | Resting membrane potentials of neurons grown onto glass:P3HT or glass.

Resting membrane potentials (means ± sem) of neurons plated on bare glass (-53.72 ± 1.36 mV, *n* = 20), glass:P3HT (-50.50 ± 1.25 mV, *n* = 27), and glass:P3HT in the presence of synaptic blockers (-54.16 ± 1.99 mV, *n* = 14). No significant difference among the groups (p = 0.1348, one-way ANOVA).

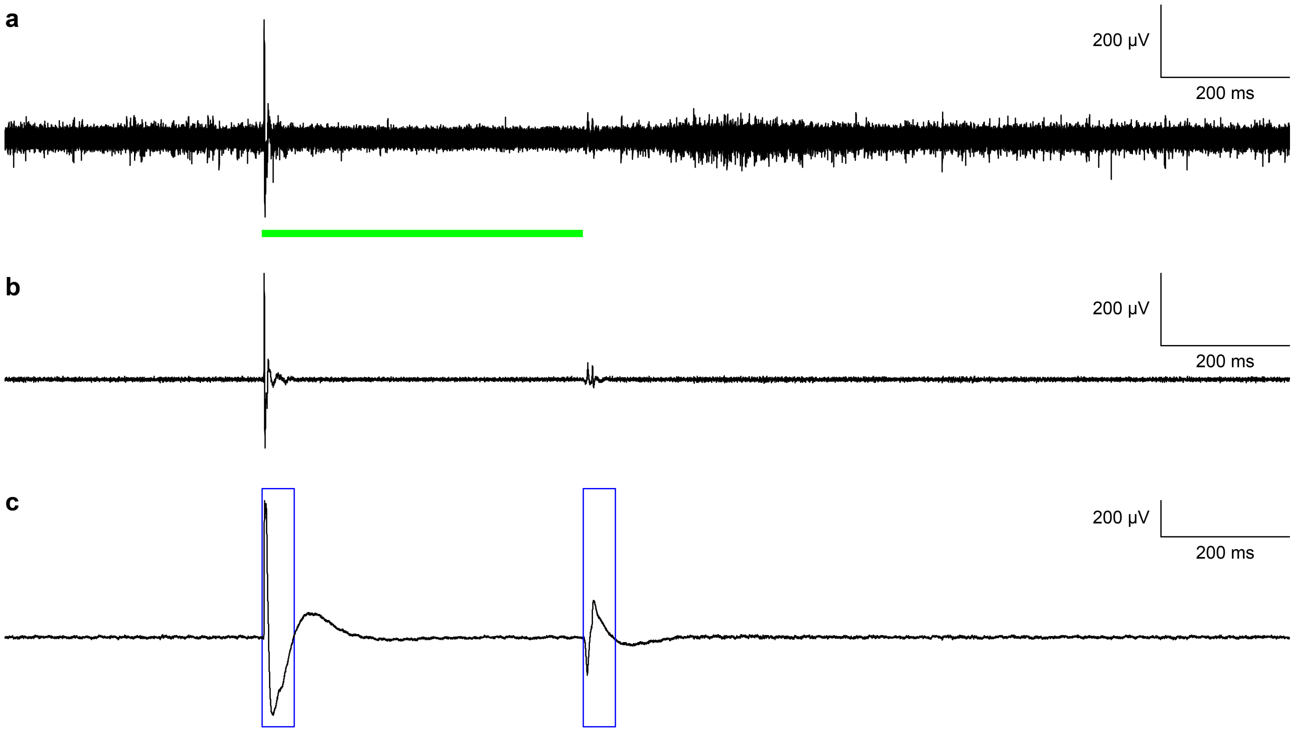

Supplementary Figure 4 | Light evoked artifacts in MEA recordings in response to light stimulation.

a. Representative trace (overlay of 20 sweeps) recorded from one MEA electrode upon illumination (500 ms, green bar) and filtered from 200 Hz to 3000 Hz.

b. Average representative trace highlighting light stimulation artifacts.

c. High-pass filtering at 10 Hz of the representative average trace showing the extent of the artifacts and of the excluded bins (blue boxes).

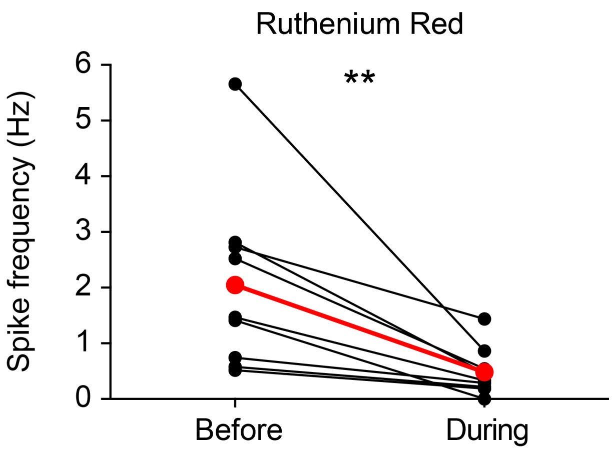

Supplementary Figure 5 | Spike reduction in primary hippocampal neurons is not affected by TRPV channels.

Primary hippocampal neurons plated on P3HT substrates were recorded under current clamp to investigate spontaneous action potential firing in extracellular medium containing 10 μM Ruthenium Red. The cells were exposed to multiple repetitions of green light pulses (500ms, 16mW/mm^2). A statistically significant reduction of firing rate ($p = 0.0014$) was observed during the light pulse.

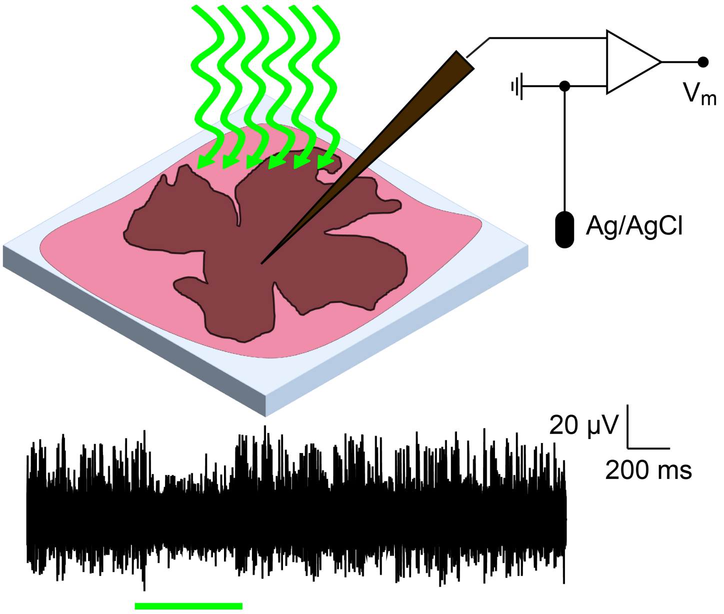

Supplementary Figure 6 | Recordings with metal electrodes of retinal ganglion cells activity on pristine P3HT films.

Schematic representation of the experimental set-up and representative trace (overlay of 20 sweeps) recorded upon illumination (green bar; 500 ms, 15 mW/mm^2).

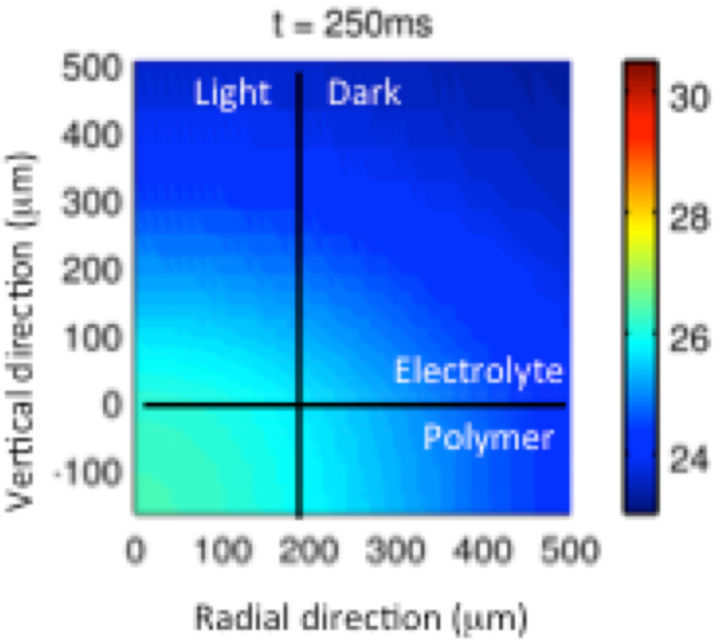

Supplementary Figure 7 | Spatial distribution of the temperature profile during illumination. The spatial distribution of the temperature profile during illumination along the radial and the axial directions (in μm), during a 250 msec pulse of 57 mW/mm^2, as obtained from a numerical simulation based on the heat diffusion equation. Notice that the temperature increase is uniform along the polymer thickness, in the order of 150 nm.

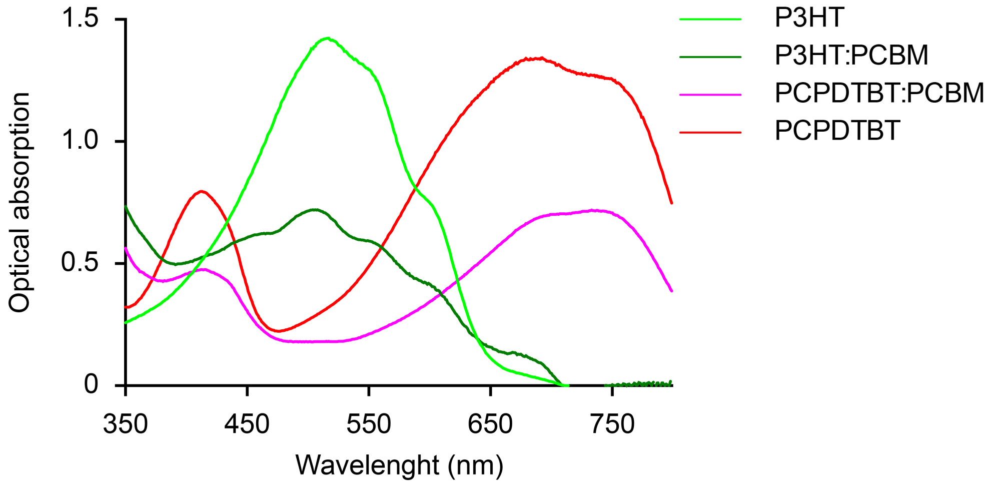

Supplementary Figure 8 | Optical absorbance of the polymeric films used in the work. Optical absorption spectra of P3HT, P3HT:PCBM, PCPDTBT, and PCPDTBT:PCBM thin films, deposited on glass coverslips. For scale comparison, the absorption spectrum of P3HT:PCBM was increased five times. Illumination peak was set at 530-540 nm for P3HT and P3HT:PCBM, and at 780 nm for PCPDTBT and PCPDTBT:PCBM.

Supplementary Table 1

	Neuron	Injected Current (pA)	Firing at Light Off (Hz)	Firing at Light On (Hz)	Firing Reduction (%)
Glass:P3HT	1	10	18.500000	3.250000	-82.432430
	2	10	13.333330	12.500000	-6.249980
	3	10	8.588235	0.088235	-98.972610
	4	20	9.400000	0.050000	-99.468090
	5	10	5.250000	0.500000	-90.476190
	6	10	7.857143	0.000000	-100.000000
	7	10	11.764710	0.882353	-92.500000
	8	30	10.000000	0.833333	-91.666670
	9	20	9.500000	2.750000	-71.052630
	10	50	3.000000	3.000000	0.000000
	11	20	6.666667	1.388889	-79.166670
	12	150	5.800000	0.300000	-94.827590
	13	20	2.000000	2.000000	0.000000
	14	200	7.000000	6.000000	-14.285710
Glass	1	10	4.500000	4.375000	-2.77778
	2	15	5.000000	5.000000	0.00000
	3	20	11.238940	7.079646	-37.00789
	4	15	9.333333	6.000000	-35.71428
	5	40	7.000000	5.500000	-21.42857
	6	100	3.000000	4.666667	55.55557
	7	40	2.750000	2.375000	-13.63636
	8	20	2.200000	2.800000	27.27273
	9	50	2.166667	3.250000	49.99998
	10	200	6.333333	4.333333	-31.57895
	11	100	5.500000	5.750000	4.54545
	12	20	11.333330	7.500000	-33.82351
	13	200	3.333333	4.000000	20.00001
	14	100	6.500000	5.500000	-15.38462
	15	100	14.000000	6.000000	-57.14286
	16	40	2.000000	3.000000	50.00000
	17	150	11.500000	7.000000	-39.13043
	18	20	2.250000	1.750000	-22.22222
	19	20	2.000000	3.250000	62.50000
